# Extracellular production of a thermostable *Cellvibrio* endolytic β-agarase in *Escherichia coli* for agarose liquefaction

**DOI:** 10.1186/s13568-023-01551-w

**Published:** 2023-05-05

**Authors:** Hee Kyoung Lee, Won Young Jang, Young Ho Kim

**Affiliations:** Laboratory of Immunobiology, School of Life Science and Biotechnology, College of Natural Sciences, Kyungpook National University, 80 Daehak-ro, Buk-gu, Daegu, Republic of Korea

**Keywords:** Freshwater agar-degrading *Cellvibrio*, Neoagarotetraose, Neoagarohexaose, Recombinant his-tagged enzyme, Sephadex G-15 column chromatography, Thermostable endolytic GH16B β-agarase

## Abstract

Four GH16 family β-agarases (GH16A, GH16B, GH16C, and GH16D), originated from an agarolytic bacterium *Cellvibrio* sp. KY-GH-1, were expressed in an *Escherichia coli* system and their activities were compared. Only GH16B (597 amino acids, 63.8 kDa), with N-terminal 22-amino acid signal sequence, was secreted into the culture supernatant and demonstrated a robust endolytic agarose hydrolyzing activity for producing neoagarotetraose (NA4) and neoagarohexaose (NA6) as end products. The optimal temperature and pH for the enzyme activity were 50 °C and 7.0, respectively. The enzyme was stable up to 50 °C and over a pH range of 5.0–8.0. The kinetic parameters, including *Km*, *Vmax*, *kcat*, and *kcat/Km*, of GH16B β-agarases for agarose were 14.40 mg/mL, 542.0 U/mg, 576.3 s^−1^, and 4.80 × 10^6^ s^−1^ M^−1^, respectively. The addition of 1 mM MnCl_2_ and 15 mM tris(2-carboxyethyl)phosphine enhanced the enzymatic activity. When agarose or neoagaro-oligosaccharides were used as substrates, the end products of enzymatic catalysis were NA4 and NA6, whereas agaropentaose was produced along with NA4 and NA6 when agaro-oligosaccharides were used as substrates. Treatment of 9%[w/v] melted agarose with the enzyme (1.6 µg/mL) under continuous magnetic stirring at 50 °C for 14 h resulted in efficient agarose liquefaction into NA4 and NA6. Purification of NA4 and NA6 from the enzymatic hydrolysate (9%[w/v] agarose, 20 mL) via Sephadex G-15 column chromatography yielded ~ 650 mg NA4/~ 900 mg NA6 (i.e., ~ 85.3% of the theoretical maximum yield). These findings suggest that the recombinant thermostable GH16B β-agarase is useful for agarose liquefaction to produce NA4 and NA6.

**Graphical Abstract:**

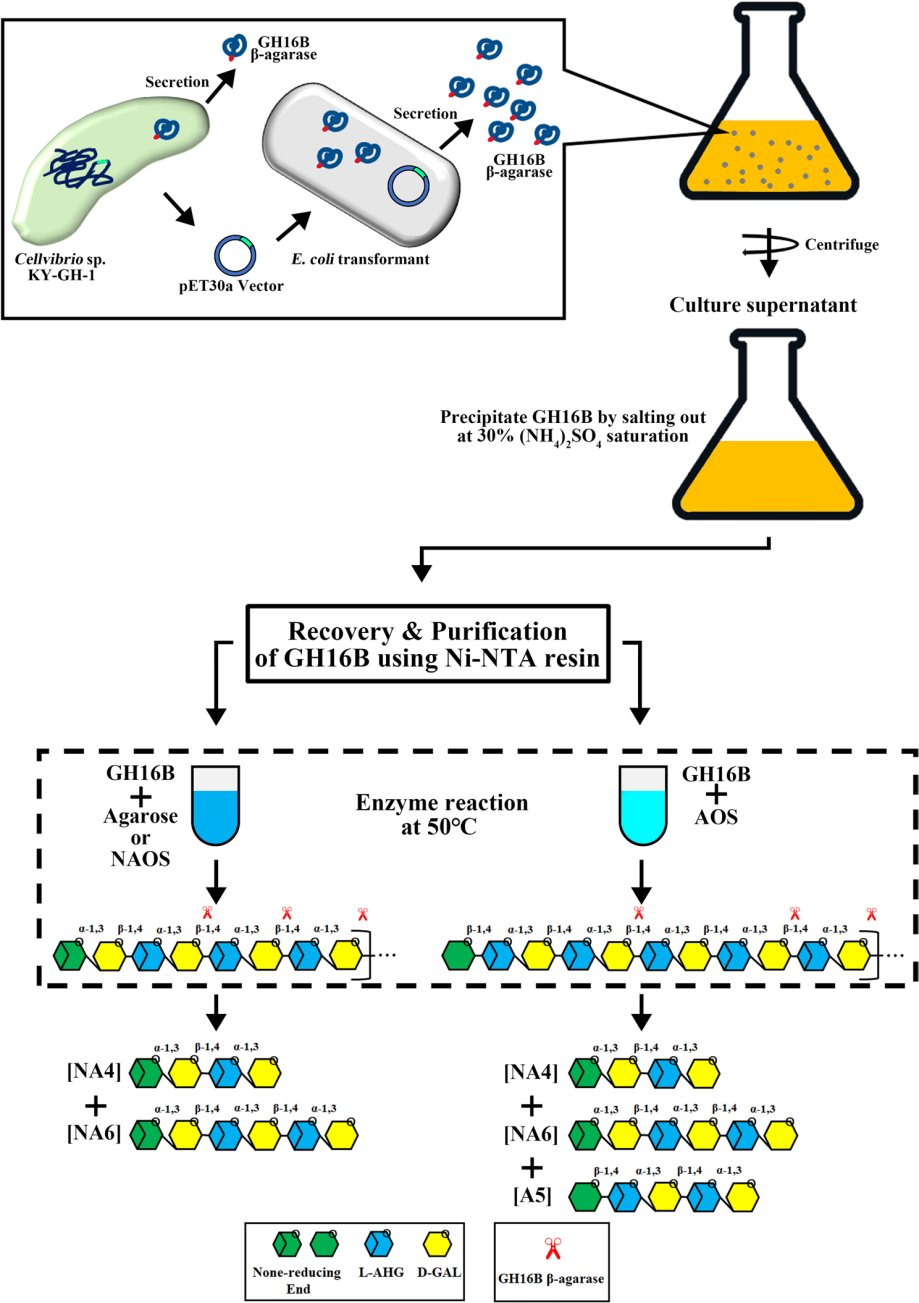

## Key points

- Thermostable GH16B β-agarase was expressed extracellularly in *Escherichia coli*.
- Complete enzymatic hydrolysis into NA4 and NA6 was achieved using 9%[w/v] melted agarose.
- The enzyme is useful for producing NAOSs (e.g., NA4 and NA6) via agarose liquefaction.
- NA4 and NA6 in the hydrolysates were purified via Sephadex G-15 column chromatography.

## Introduction

Agar derived from marine red algae, such as *Gelidium*, *Gracilariais*, and *Gelidiella*, contains two major components agarose and agaropectin. Agarose is a non-ionic linear polysaccharide consisting of the repeating unit of neoagarobiose disaccharide, in which 3,6-anhydro-α-l-galactose is α-1,3-linked to β-d-galactose and resulting neoagarobiose is linked by β-1,4 glycosidic bond between β-d-galactose and 3,6-anhydro-α-l-galactose (Chi et al. 2012; Duckworth and Yaphe 1971; Hehemann et al. 2012). Agaropectin has the same repeating units heavily modified with anionic groups such as –OSO_3_−, –OCH_3_, glucuronate, or pyruvate residues (Chi et al. 2012; Duckworth and Yaphe 1971; Mandal et al. 2023; Wang et al. 2012). The difference in their ionic charges has been applicated to the separation of agarose from agaropectin. Agar is commonly used in microbial culture media and food industries as a stable gelling agent and is resists to microbial degradation.

Several marine bacteria and few nonmarine bacteria are known to degrade agar into the monomeric sugars L-AHG and D-Gal for use as the sole carbon source (Bannikova et al. 2008; Fu and Kim 2010; Jahromi and Barzkar 2018; Rhee et al. 2010). For complete agar hydrolysis into monomers, an agarolytic bacterium capable of assimilating agar as a carbon source commonly produces a combination of various agarases. Agarases are classified into α-agarase (EC 3.2.1.158) and β-agarase (EC 3.2.1.81), which cleave the α-1,3-glycosidic and β-1,4-glycosidic linkages, respectively (Fu and Kim 2010; Jahromi and Barzkar 2018). The most commonly reported β-agarases are endo-acting agarases that hydrolyze agarose into neoagaro-oligosaccharides (NAOSs). There are few reports on α-agarases, which hydrolyze agarose into agaro-oligosaccharides (AOSs). Bacterial agarases are classified into different glycoside hydrolase (GH) families based on similarities in their amino acid (aa) sequences. β-Agarases belong to the families GH16, GH50, GH86, and GH118 (Cantarel et al. 2009; Chi et al. 2012; Michel et al. 2006), whereas α-agarases belong to the families GH96 and GH117 (Flament et al. 2007; Ha et al. 2011; Jang et al. 2021; Lee et al. 2019). Each agarose family degrades agarose in a distinct manner. Endo-acting β-agarases from the GH16, GH86, and GH118 families degrade agarose into neoagarotetraose (NA4) and neoagarohexaose (NA6), NA6 and neoagarooctaose (NA8), and NA8 and neoagarodecaose (NA10), respectively. The β-agarase members of GH50 family degrade agarose via exo-acting activity or a combination of exo- and endo-acting activities to produce neoagarobiose (NA2) (Kwon et al. 2020; Han et al. 2016), NA4 (Chen et al. 2018), or NA2 and NA4 (Li et al. 2015; Liang et al. 2017) as end products. GH96 α-agarases hydrolyze agarose into agarotetraose (Flament et al. 2007; Lee et al. 2019), whereas GH117 α-neoagarobiose hydrolases (α-NABH) cleave NA2 into L-AHG and D-Gal (Ha et al. 2011; Jang et al. 2021).

Several health benefits of agarose degradation products have recently attracted attention. These include antioxidant activities of agarotriose (A3) (Chen and Yan 2005) and NAOSs containing NA6/NA8 (Xu et al. 2018); prebiotic activities of A3 (Yun et al. 2021) and NAOSs containing NA4–NA10 (Hu et al. 2006); anti-inflammatory activity of NA4 in mouse macrophage RAW264.7 cells (Wang et al. 2017); antidiabetes and antiobesity activities of NAOSs comprising NA4/NA6 (Hong et al. 2017); dendritic cell-stimulating effect of NA6, leading to augmenting antitumor activity in NK cells ((Lee et al. 2017b); and skin moisturizing activity of NA2 with a good hygroscopic ability (Kobayashi et al. 1997). Furthermore, in a mouse model, oral administration of NA4 could modulate the gut microbiota, providing health benefits (Zhang et al. 2017). In terms of L-AHG bioactivities, anti-inflammatory activity in mouse macrophage RAW264.7 cells (Yun et al. 2013), skin-whitening activity which could inhibit melanin production in B16F10 murine melanoma cells (Yun et al. 2013; Kim et al. 2017b), and antitumor activity which was exerted by inducing apoptotic cell death against HCT116 human colon cancer cells (Yun et al. 2021) have been reported.

In this context, various studies have focused on the characterization of novel agarolytic enzymes, which are obtained as recombinant enzymes or purified enzymes from agarolytic bacteria, as well as the development of individual enzymatic processes to produce L-AHG, NA2, NA4, NA6, and NAOSs from agarose (Chen et al. 2021; Park et al. 2020). These processes, however, can be improved for enhanced enzymatic saccharification of agarose into L-AHG and D-Gal. Further research on GH16 family β-agarases is urgently needed, considering that the action of these enzymes can efficiently liquefy agarose to yield NAOSs with low degrees of polymerization (DPs). These enzymes can produce NA2 through their combination with exolytic GH50 β-agarase as well as L-AHG and D-gal through their combination with exolytic GH50 β-agarase and α-NABH.

By sequencing the entire genome of a freshwater agarolytic bacterium *Cellvibrio* sp. KY-GH-1 (KCTC 13629BP), we recently reported the genes encoding agarases (Kwon et al. 2019). The strain was predicted to possess a 77-kb agarase gene cluster encoding four endolytic β-agarases (GH16A, GH16B, GH16C, and GH16D). However, it remains unknown which isozyme possesses the most prominent endolytic GH16 β-agarase activity for agarose liquefaction and NAOS production.

In this study, an *Escherichia coli* expression system using pET-30a vector was employed to obtain four recombinant His-tagged GH16 family β-agarases (GH16A, GH16B, GH16C, and GH16D) from *Cellvibrio* sp. KY-GH-1, and their enzymatic activities were compared. The isozyme with the highest endolytic β-agarase activity and its efficiency for NA4/NA6 production via agarose liquefaction were also investigated.

## Materials and methods

### Materials

Agarose, Micro BCA protein assay kit, Ni-NTA resin, and PageRuler prestained protein ladder were purchased from ThermoFisher Scientific (Rockford, IL, USA). Ammonium sulfate, chloramphenicol, complete Freund’s adjuvant, Coomassie brilliant blue (CBB) R-250, 3,5-dinitrosalicylic acid (DNS), ethylenediamine tetraacetic acid (EDTA), d-galactose, incomplete Freund’s adjuvant, isopropyl β-d-1-thiogalactopyranoside (IPTG), kanamycin, naphthoresorcinol, sodium dodecyl sulfate (SDS), and tris(2-carboxyethyl)phosphine (TCEP) were obtained from Sigma-Aldrich (St. Louis, MO, USA). Restriction enzymes and T4 ligase were obtained from Roche (Basel, Switzerland). The pET-30a vector for expressing C-terminal 6× His-tagged protein and Immobilon-P membrane were purchased from EMD Millipore Corporation (Temecula, CA, USA). A standard NAOS mixture (NA2–NA18) was provided by Dr. Sang-Hyeon Lee (Lee et al. 2008). An AOS mixture was prepared by mild acid hydrolysis of 1%[w/v] agarose as previously described (Kwon et al. 2020). The standards (NA2, NA4, and NA6) were obtained from Carbosynth Ltd. (Berkshire, UK). *E. coli* BL21 (DE3) pLysS was obtained from Novagen (Madison, WI, USA).

### Expression of recombinant GH16 β-agarases using the *E. coli* expression system

Four GH16 β-agarase genes (encoding GH16A [GenBank accession no. WP_151030829.1], GH16B [GenBank accession no. WP_151030830.1], GH16C [GenBank accession no. QEY14919.1], and GH16D [GenBank accession no. WP_151030839.1]) in the genomic DNA of *Cellvibrio* sp. KY-GH-1 strain were amplified using polymerase chain reaction (PCR) with the following primers: NdeI-forward primers (5′-GCGGCATATG-AAAAAAATCACTTCATGTA-3′ for GH16B, 5′-GCGGCATATGCAGTTAATAGATAATAAAGAG-3′ for GH16C, 5′-GCGGCATATGCGTATTCGCTCACCTTG- 3′ for GH16D, and 5′-CGCATATGGCCGATTGGG-ATTCAGTTCC-3′ for GH16B without a signal sequence); BamHI-forward primer (5′-CGGGATCCATG-AAAAAGCATATTTCATGCTG-3′ for GH16A); XhoI-reverse primers (5′-CTAACTCGAGGGGTATCAA-TTCAAACTTG-3′ for GH16B and GH16B without a signal sequence, 5′-CGCGCTCGAGGGGTACTAATT-CAAATTTATCC-3′ for GH16C, and 5′-CGCGCTCGAGATTTACTGGCACAAACTCAATG-3′ for GH16D); and HindIII-reverse primer (5′-CGAAGCTTGAGTGAGCCAACTCGAGTAA-3′ for GH16A). The PCR products were digested with appropriate restriction enzyme sets before being ligated into the pET-30a vector with T4 ligase. The recombinant pET-30a plasmids were transformed into *E. coli* BL21 (DE3) pLysS. After overnight incubation at 30 °C on Luria broth (LB) plates supplemented with 50 µg/mL chloramphenicol and 25 µg/mL kanamycin, the transformants were selected.

The expression of each recombinant β-agarase in the transformant was induced as previously described (Jang et al. 2021) with minor modifications. Briefly, each transformant was cultured at 25 °C with shaking in LB containing 50 µg/mL chloramphenicol and 25 µg/mL kanamycin. When optical density at 600 nm (OD_600_) of the culture reached 0.5–0.6, 0.5 mM IPTG was added to induce protein expression. Then, the culture was incubated at 20 °C for 20 h.

### Zymogram plate assay for β-agarase activity determination

*Escherichia coli* BL21 (DE3) pLysS transformants harboring the recombinant pET-30a plasmid containing GH16A, GH16B, GH16C, or GH16D were spotted onto LB agarose plates (1.5%[w/v] agarose, 50 µg/mL chloramphenicol, 25 µg/mL kanamycin, and 0.1 mM IPTG) and then incubated for 3 days at 37 °C. The plates were stained with Lugol’s iodine solution at 25 °C.

### Sequence analysis and construction of phylogenetic tree

The aa sequences of GH16 family β-agarases were obtained for analyzing their similarity via a BLAST search of the NCBI database (http://www.ncbi.nlm.nih.gov). Multiple alignment of the aa sequences was performed using Clustal X program (Larkin et al. 2007), and conserved residues were highlighted using Clustal X color scheme, as previously described (Kwon et al. 2020). Using UPGMA, an unrooted phylogenetic tree of GH16 family members was constructed based on aa sequence similarities (Sneath and Sokal 1973). SignalP version 5.0 was used to predict the location of the N-terminal signal sequence of GH16B β-agarase (Almagro Armenteros et al. 2019).

### Cell lysate preparation, protein quantitation, sodium dodecyl sulfate–polyacrylamide gel electrophoresis (SDS–PAGE), and western blot analysis

To identify and localize the recombinant enzyme proteins produced by the transformants, cell cultures were centrifuged at 8000 rpm for 20 min, and culture supernatants (CS) and cell pellets were separated. Cell pellets were suspended in 40 mM Tris-HCl buffer (pH 8.0), sonicated using 50 short bursts of 2 s each, extracted at 4 °C for 30 min, and fractionated into three intracellular fractions (total, soluble, and pellet [insoluble inclusion]), as previously described (Jun et al. 1996). After quantifying protein concentrations of cell lysate using Micro BCA protein assay kit, the same portion of each fraction was subjected to 8% SDS–PAGE. By staining the gels with CBB R-250, recombinant enzymes were visualized. For western blot analysis, equivalent amounts of *E. coli* cell lysates and culture supernatants were resolved by SDS–PAGE and then electrotransferred onto Immobilon-P membranes as previously described (Lee et al. 2017a). ECL Prime Western Blotting Kit (Amersham, Arlington Heights, IL, USA) was used to detect the proteins.

### Purification of GH16B β-agarase

The supernatant of transformed *E. coli* cultures (300 mL), obtained following centrifugation (8000 rpm for 20 min, was mixed with ammonium sulfate (30% saturation), and the mixture was stirred overnight at 4 °C to precipitate the His-tagged GH16B β-agarase. The precipitate was collected via centrifugation (20,000 rpm for 20 min), dissolved in 10 mL of 10 mM Tris-HCl buffer (pH 7.5), and dialyzed at 4 °C for 3 h. The dialyzed recombinant protein was purified via immobilized metal affinity chromatography with Ni-NTA resin. The purified enzyme was lyophilized to dryness.

### Production of a polyclonal antibody against recombinant GH16B β-agarase

To prepare mouse polyclonal antibody specific for GH16B β-agarase, purified recombinant GH16B β-agarase solution (120 µg/300 µL of phosphate-buffered saline) was mixed with an equal volume of complete Freund’s adjuvant, and the mixture (20 µg/100 µL) was intraperitoneally injected into mice. A purified GH16B β-agarase solution was mixed with an equal volume of incomplete Freund’s adjuvant and injected intraperitoneally into the mice every 10 day for secondary, tertiary, and quaternary immunization. Five days after quaternary immunization, the mice were bled. C57BL/6J male mice (6-weeks-old) were purchased from the JA Bio (Suwon-si, Gyeonggi-do, Korea) and maintained at the Animal Resources Center in Kyungpook National University (Daegu, Korea) under specific pathogen-free conditions.

### Agarase activity assay

GH16B β-agarase activity was measured as previously described (Miller 1959). One hundred microliters of the enzyme solution was mixed with an equal volume of 0.8%[w/v] melted agarose in an appropriate buffer. The content of reducing sugars in the reaction mixture was determined colorimetrically using DNS reagent after 30 min of incubation at 50 °C. The amount of enzyme that produced reducing power equivalent to 1 µmol of D-Gal per min was defined as one unit of enzymatic activity.

The effect of temperature on the enzymatic activity was measured using 50 mM Mcllvaine buffer buffer (pH 7.0) for 30 min over a temperature range of 25–70 °C. The effect of pH on the enzymatic activity was measured using 50 mM McIlvaine (pH 4.0–7.0) and 50 mM Tris–HCl (pH 7.0–10.0) buffer. For temperature and pH stability assays, aliquots of enzyme were preincubated over a range of temperatures and pH for indicated time, and the relative enzymatic activity was measured. The kinetic parameters of GH16B β-agarase were determined using a known amount of the enzyme and substrate solution (1–5 mg/mL agarose in 50 mM Mcllvaine buffer, pH 7.0). The Lineweaver–Burk equation, generated via GraphPad Prism 8 statistical package (GraphPad Software Inc, Boston, MA, USA), was used to calculate *Km* and *Vmax* values. Effects of metal ions (CaCl_2_, CuSO_4_, KCl, MgCl_2_, MnCl_2_, MnSO_4_, and NaCl), reducing agent (TCEP), and a chelator (EDTA) on the enzymatic activity were examined by adding these reagents at indicated concentrations to the standard assay mixture.

### Thin-layer chromatography (TLC)

TLC was performed for GH16B β-agarase-catalyzed hydrolysates of agarose, NAOS, or AOS on Silica Gel 60 aluminum plates coated with fluorescent indicator F254 (Merck, Darmstadt, Germany), which were developed with *n*-butanol-ethanol-H_2_O (3:2:2[v/v]). To detect oligosaccharides, a visualization solution (0.2%[w/v] naphthoresorcinol, 10%[v/v] H_2_SO_4_, ethanol) was sprayed on the plates, followed by heating at 80 °C. L-AHG (Jang et al. 2021), the NAOS mixture (NA2–NA18) (Lee et al. 2008), and commercial NA2, NA4, and NA6 were used as standards.

### Analyses of the enzymatic hydrolysates using matrix-assisted laser desorption/ionization time-of-flight/time-of-flight mass spectrometry (MALDI–TOF/TOF MS)

To analyze the hydrolytic products of agarose or AOS produced via enzymatic reaction, MALDI–TOF/TOF MS analysis was performed in the positive ion reflectron mode using Autoflex max (Bruker Daltonics, Billerica, Massachusetts, USA), as described elsewhere (Lee et al. 2014). The enzymatic reaction mixture was spotted onto a stainless steel target plate, followed by the addition of 0.5 µL of 50% acetonitrile in which excessive 2,5-dihydroxybenzoic acid was dissolved, and air-dried. Each acquired spectrum represents the combined signal from 500 laser shots at four random locations on the spot for a total of 2000 laser shots using a 2 kHz laser. The laser attenuator offset and range were set at 18% and 40%, respectively, with the laser focus set at 36%. Mass spectra were recorded over an m/z range of 500–1500. To obtain high-resolution data, the detector sampling rate was set at a maximum of 2.50 giga samples/s, and the detector gain was set at 6.2×. flexAnalysis software (version 3.4; Bruker Daltonics) was used to process the raw MS data.

### Purification of NA4 and NA6 via size-exclusion column chromatography

To purify NA4 and NA6 from the hydrolytic products of agarose, 9%[w/v] melted agarose (20 mL) was treated with GH16B β-agarase under optimal conditions (1 mM MnCl_2_ and 10 mM TCEP, 50 mM Mcllvaine buffer, pH 7.0, 50 °C) with continuous magnetic stirring, followed by freeze–drying. The dried samples were dissolved in deionized (DI) water and fractionated via size-exclusion chromatography using Sephadex G-15 column (I.D. 1.3 × 120 cm). Two milliliters of fractions eluting in DI water were collected. TLC was used to identify fractions containing NA4 and NA6, which were then collected and lyophilized to dryness.

### Statistical analyses

Unless otherwise specified, the data represent at least three independent experiments and are expressed as the mean ± standard deviation (n ≤ 3). Statistical analyses were performed using Student’s *t*-test to compare the data between two groups, whereas one-way analysis of variance followed by Dunnett’s multiple comparison test was used to compare three or more groups. *P* values of < 0.05 were considered to indicate statistical significance. Statistical analysis was conducted using SPSS Statistics version 23 (IBM, Armonk, NY, USA).

## Results

### Production of four *Cellvibrio* sp. KY-GH-1-derived GH16 family β-agarases as recombinant proteins with C-terminal His-tag using the *E. coli* expression system

**Fig. 1:**
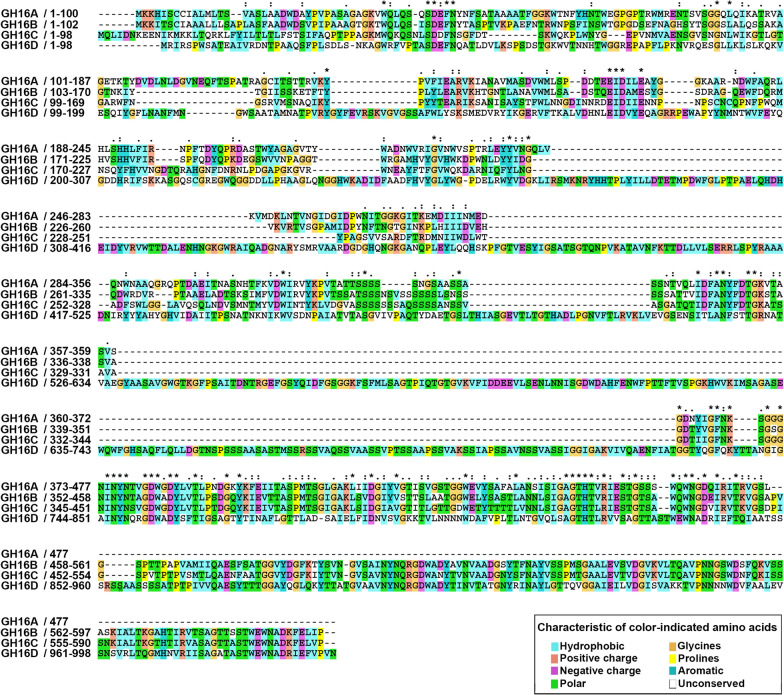
Comparison of aa sequences of GH16A, GH16B, GH16C, and GH16D β-agarases from *Cellvibrio* sp. KY-GH-1. Amino acids are displayed as one-letter abbreviations after the alignment for maximal identity using Clustal X (Larkin et al. 2007). Identical residues among the four sequences are indicated by asterisks above the column; residues are highlighted using Clustal X color scheme. Deletions are indicated by dashes

A previous study on whole genome sequence analysis of an agarolytic bacterium, *Cellvibrio* sp. KY-GH-1, revealed four GH16 β-agarase genes (GH16A, GH16B, GH16C, and GH16D) (Kwon et al. 2019). As shown in Fig. 1, GH16A has a predicted open reading frame (ORF) encoding GH16A β-agarase (52.0 kDa, 477 aa), GH16B has a predicted ORF encoding GH16B β-agarase (63.8 kDa, 597 aa), and GH16C has a predicted ORF encoding GH16C β-agarase (64.3 kDa, 590 aa). In contrast, GH16D has a predicted ORF encoding GH16D β-agarase (108.1 kDa, 998 aa), the heaviest isozyme among the four GH16 β-agarases. The GH16B β-agarase sequence shared 51.7%, 52.0%, and 41.0% identity with that of GH16A, GH16C, and GH16D β-agarases, respectively, indicating low homology among the four GH16 β-agarases in *Cellvibrio* sp. KY-GH-1.

**Fig. 2:**
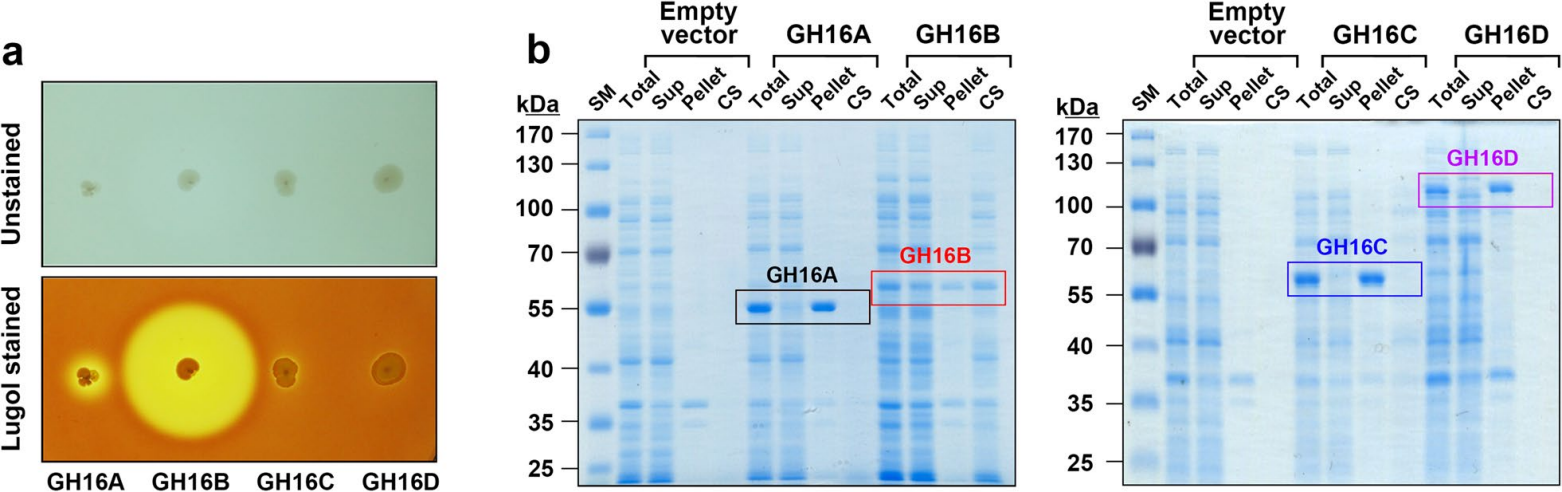
Detection of recombinant His-tagged GH16A, GH16B, GH16C, and GH16D β-agarases expressed in *Escherichia coli* on LB agarose plates and on SDS-polyacrylamide gels. **a** Transformants expressing individual recombinant His-tagged β-agarases were spotted onto LB agarose plates (1.5%[w/v] agarose, 0.1 mM IPTG, 50 µg/mL chloramphenicol, and 25 µg/mL kanamycin) and incubated for 3 days at 37 °C. To visualize the enzymatic activity as a clear zone, plates were stained with Lugol’s iodine at 25 °C. **b** Total, soluble supernatant (Sup), and insoluble inclusion (Pellet) intracellular fractions as well as culture supernatants (CS) as extracellular fractions were prepared from *E. coli* transformants cultured in the presence of 0.5 mM IPTG. Equivalent amounts of each portion were subjected to SDS–PAGE. Representative results are shown; two additional experiments yielded similar results

By staining individual colonies expressing one of the four GH16 β-agarases with Lugol’s iodine solution, we identified the GH16 family β-agarase with the highest endolytic activity for agar degradation. As evidenced by a bright clear zone around the colonies, the GH16B β-agarase-expressing transformant appeared to possess the highest agarose-degrading activity (Fig. 2a). Next, we examined the intracellular soluble/insoluble ratio and extracellular secretion ratio of each recombinant enzyme. As shown in Fig. 2b, GH16A, GH16C, and GH16D β-agarases were predominantly detected in the intracellular insoluble inclusion bodies, whereas GH16B β-agarase was mostly found in culture supernatants (CS), indicating that the recombinant GH16B β-agarase was secreted out of *E. coli* cells into the culture medium.

These findings suggest that GH16B β-agarase is the major endolytic β-agarase isozyme in the agarose-degrading enzymatic machinery of *Cellvibrio* sp. KY-GH-1 and may act as an extracellular enzyme.

### Comparison of amino acid sequences of GH16B β-agarases from KY-GH-1 and other sources

**Fig. 3:**
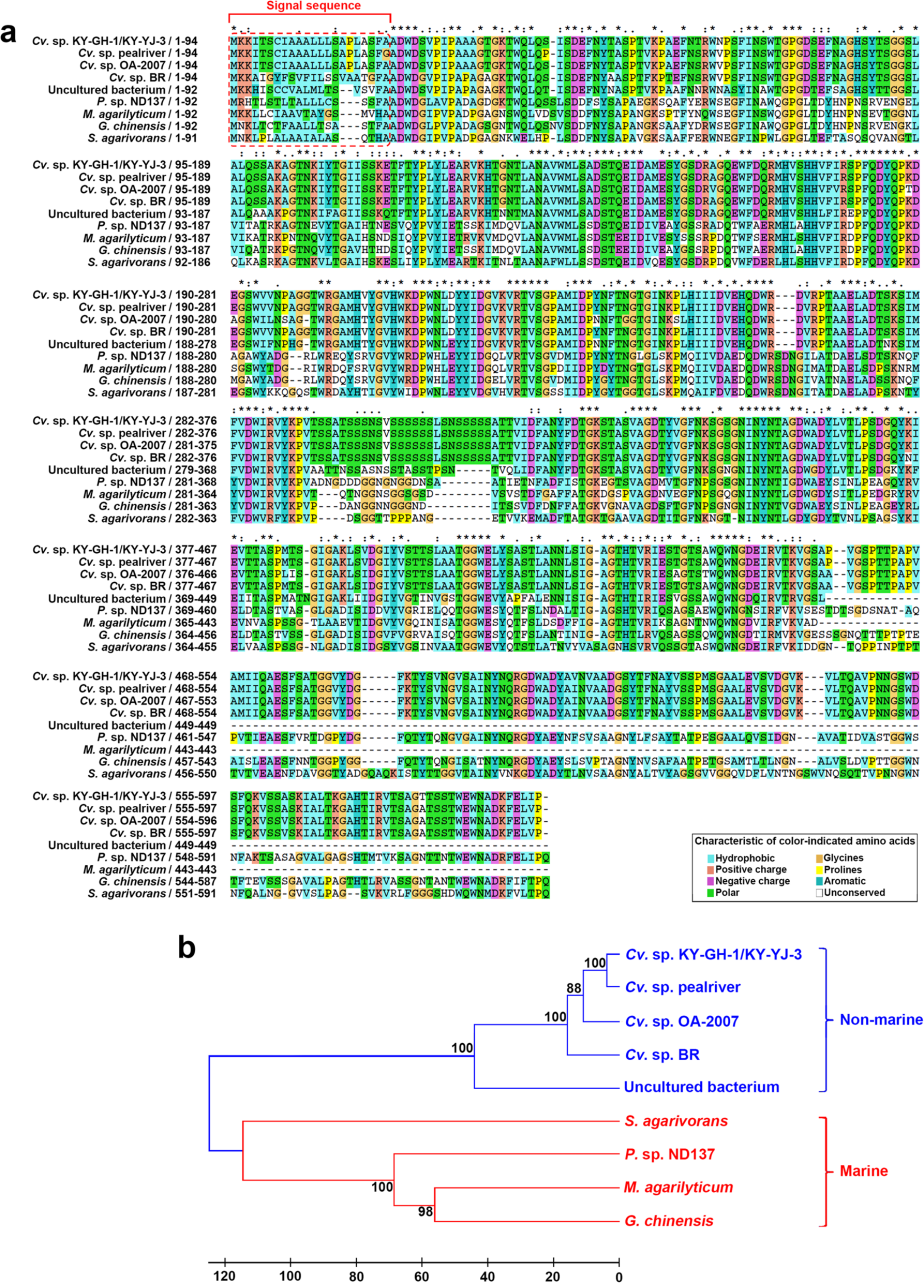
Multiple amino acid sequence alignment comparing GH16B β-agarase with nine GH16 family β-agarases available in the Genbank database and their phylogenetic relationship. **a** Amino acids are designated using one-letter abbreviations after the alignment of aa sequences using Clustal X (Larkin et al. 2007). Identical residues in all sequences are indicated by asterisks under the column; conserved substitutions are indicated by colons, semiconserved substitutions are indicated by dots, and conserved residues are highlighted using Clustal X color scheme. Deletions are indicated by dashes. The location of N-terminal 22-aa signal sequence of GH16B β-agarase was predicted using SignalP version 5.0. **b** Phylogenetic relationships between GH16B β-agarase and nine related GH16B β-agarases based on their aa sequence similarities. The unrooted phylogenetic tree was constructed using UPGMA (Sneath and Sokal 1973). Numbers at nodes represent the levels of bootstrap support

The aa sequence of GH16B β-agarase from *Cellvibrio* sp. KY-GH-1 was found to be homologous to that of other GH16 family β-agarases. As shown in Figs. 3a and 597 aa of KY-GH-1 GH16B β-agarase contained a putative N-terminal signal sequence (22 aa). The GH16B β-agarase homologs from *Cellvibrio* sp. pealriver (GenBank accession no. WP_049629425.1) (Xie et al. 2017), *Cellvibrio* sp. OA-2007 (Genbank accession no. WP_062064993.1) (Syazni et al. 2015), and *Cellvibrio* sp. BR (GenBank accession no. WP_007640762.1) also appeared to contain the signal sequence at their N-terminal, supporting the possibility that these GH16 family β-agarases are produced in agarolytic *Cellvibrio* species as extracellular enzymes to degrade agarose. However, the overall aa sequence of *Cellvibrio* sp. KY-GH-1 GH16B β-agarase (GenBank accession no. WP_151030830.1) showed 100% identity with that of *Cellvibrio* sp. KY-YJ-3 GH16B β-agarase (GenBank accession no. WP_151030830.1). The GH16B β-agarase sequence of KY-GH-1 also shared 98.8%, 96.0%, 95.0%, and 79.6% similarity with that of nonmarine agarolytic bacteria, such as *Cellvibrio* sp. pealriver, *Cellvibrio* sp. OA-2007, *Cellvibrio* sp. BR, and uncultured bacterium (GenBank accession no. AAP49316.1), respectively, indicating a high degree of homology among *Cellvibrio* GH16B β-agarases. The GH16B β-agarase sequence of KY-GH-1 shared 53.1%, 52.1%, 50.9%, and 50.8% similarity with that of marine agarolytic bacteria, including *Pseudomonas* sp. ND137 (GenBank accession no. BAD88713.1) (Aoki and Kamei 2006), *Marinimicrobium agarilyticum* (GenBank accession no. WP_036188483.1), *Gilvimarinus chinensis* (GenBank accession no. WP_020208740.1), and *Simiduia agarivorans* (GenBank accession no. WP_015048661.1), respectively.

The rooted phylogenetic relationships of GH16B β-agarases revealed that the GH16B β-agarase of *Cellvibrio* sp. KY-GH-1/KY-YJ-3 is more closely associated with that of *Cellvibrio* sp. pealriver, *Cellvibrio* sp. OA-2007, *Cellvibrio* sp. BR, and uncultured bacterium isolated from nonmarine sources than with that of *Pseudomonas* sp. ND137, *M. agarilyticum*, *G. chinensis*, and *S. agarivorans* isolated from marine sources (Fig. 3b).

### Enzymatic characteristics of GH16B β-agarase

Because the aa sequence analysis data revealed the presence of a putative N-terminal 22-aa signal sequence in GH16B β-agarase, we decided to investigate whether the signal sequence is important for recombinant GH16B β-agarase secretion and/or solubility in the *E. coli* expression system. In this regard, *E. coli* transformants expressing GH16B β-agarase with or without the signal sequence were induced for protein expression, and the distribution of the recombinant protein in intracellular and extracellular portions was compared via western blot analysis using anti-GH16B β-agarase or anti-His-tag antibodies.

**Fig. 4:**
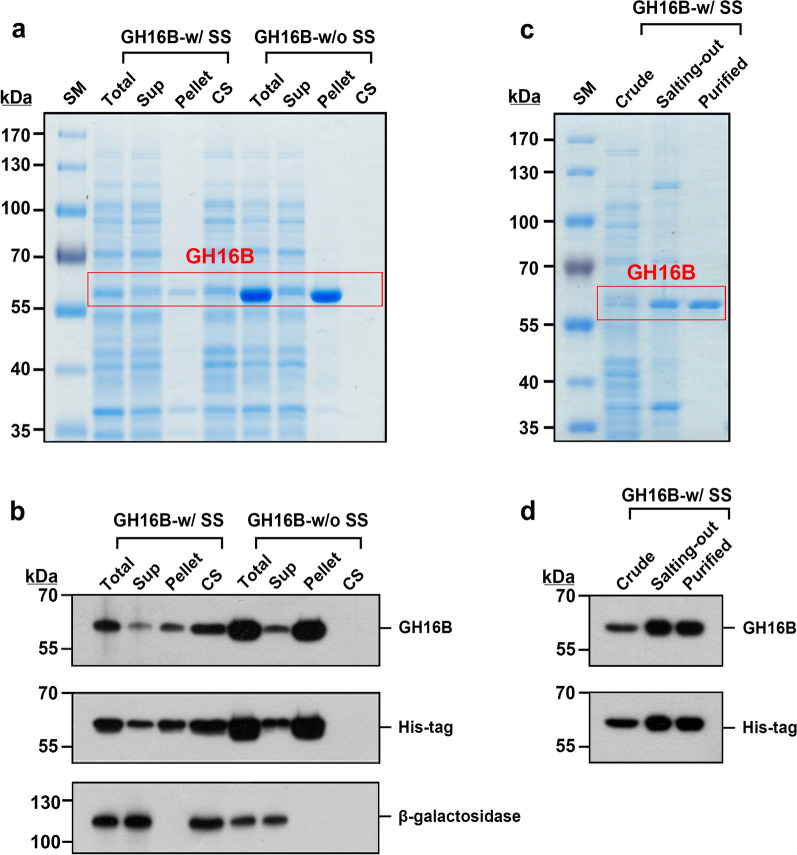
SDS–PAGE and western blot analysis of recombinant His-tagged GH16B β-agarase with or without N-terminal 22-aa signal sequence. **a**, **c** Equivalent portions of individual intracellular fractions and culture supernatants of *E. coli* transformants, and GH16B β-agarase purified from the culture supernatants using ammonium sulfate precipitation and the Ni-NTA purification system were subjected to SDS-PAGE and the gels were stained with CBB R-250 to detect protein bands. **b**, **d** For western blot analysis, proteins in the gels were electrotransferred onto Immobilon-P membranes. The membrane was then probed with antibodies specific to the protein of interest. Protein detection was performed using the ECL western blotting detection system. Lane: SM, prestained size markers; GH16B-w/SS, GH16B β-agarase with the signal sequence; GH16B-w/oSS, GH16B β-agarase without the signal sequence; crude GH16B-w/SS, enzyme in the culture supernatants (CS); salting-out GH16B-w/SS, enzyme concentrated using ammonium sulfate salting-out of the CS; purified GH16B-w/SS, enzyme purified from the concentrated GH16B-w/SS using the Ni-NTA purification system

As shown in Fig. 4a, b, GH16B β-agarase with the signal sequence was prominently detected in the extracellular portion in the soluble form; however, GH16B β-agarase without the signal sequence was not secreted and remained in the intracellular portion, where it was exclusively detected in the insoluble form. The pattern of intracellular and extracellular GH16B β-agarase distribution in the transformants, as determined via western blot analysis using anti-GH16B β-agarase and anti-His-tag antibodies, was identical. Under the same conditions, β-galactosidase, a well-known intracellular enzyme in *E. coli* (Kuboi et al. 1995), was also detected in the extracellular portion of the transformants expressing GH16B β-agarase with the signal sequence, but not in that of the transformants expressing GH16B β-agarase without the signal sequence. This suggests that expressing GH16B β-agarase with the signal sequence may cause the cells to leak some intracellular proteins, such as β-galactosidase and even GH16B β-agarase. When the His-tagged GH16B β-agarase was purified from the culture supernatant using ammonium sulfate precipitation (30% saturation), most of the proteins were excluded, except for three proteins, including GH16B β-agarase (63.8 kDa) and unknown proteins (~ 37.5 kDa and ~ 120 kDa). Further, the Ni-NTA purification resulted in the detection of a single band on 8% SDS–PAGE, with limited background contaminants, indicating the isolation of the recombinant protein with high purity (Fig. 4c, d).

**Fig. 5:**
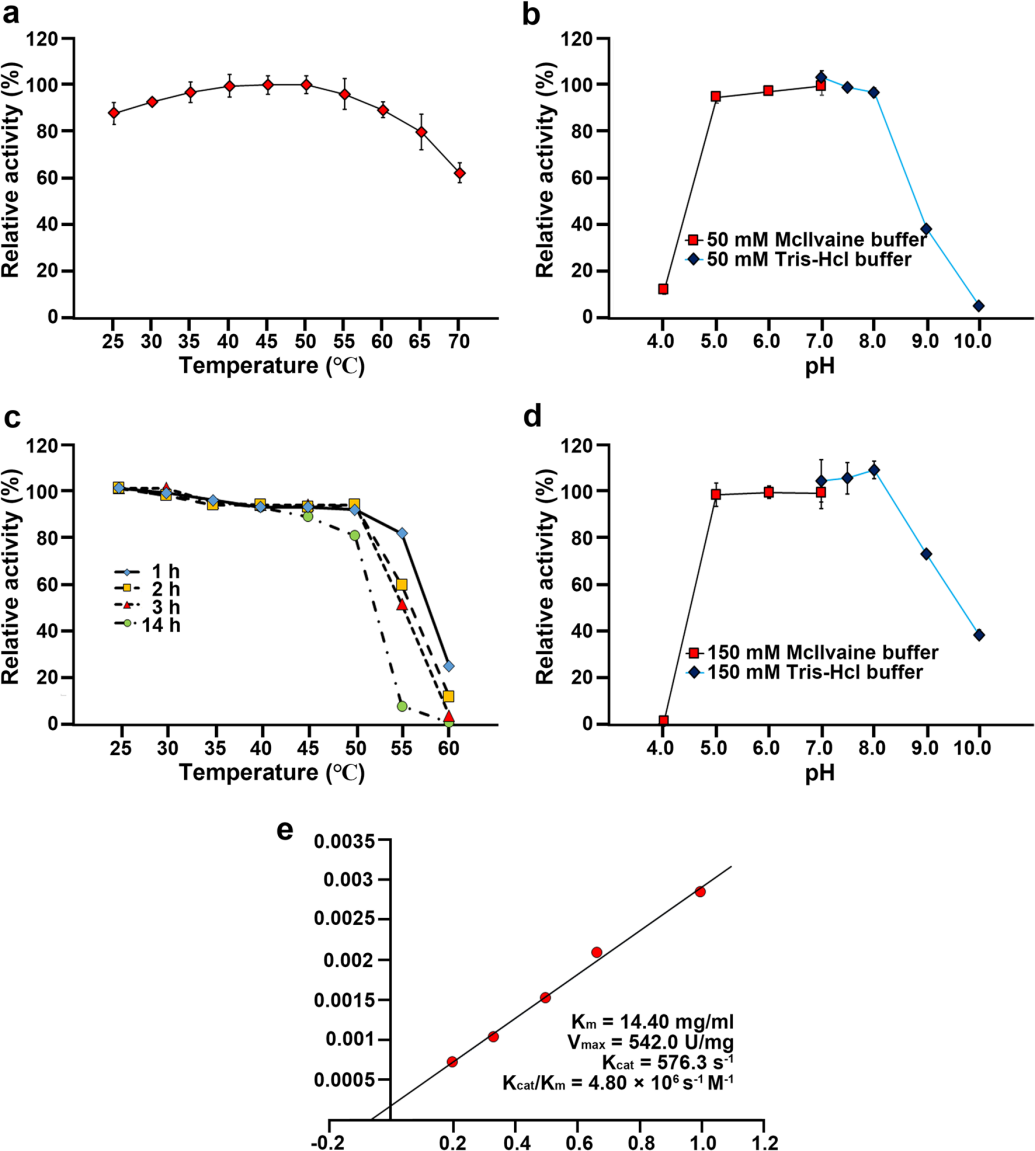
Biochemical characteristics of GH16B β-agarase. **a**, **b** The optimal temperature and pH of the enzymatic activity were determined using 0.4%[w/v] agarose and 0.8 µg/mL of the purified enzyme. Each value is expressed as the mean ± SEM (n = 3; three replicates per independent experiment). **c**, **d** Temperature and pH stabilities of the purified enzyme were measured following incubation for the indicated time at various temperatures and incubation for 4 h at various pH values. Data are expressed as the mean ± SEM (n = 3; three replicates per independent experiment). **e** Lineweaver‒Burk plot was used to determine *Km* and *Vmax* values of the purified enzyme based on indicated concentrations of the substrate (agarose) and enzyme. Representative results are shown; two additional experiments yielded similar results

The enzymatic properties of recombinant GH16B β-agarase were investigated to assess the efficacy in agarose liquefaction and subsequent NAOS production with low DPs. The highest activity was detected at 50 °C (Fig. 5a). Further, ≥ 80% of the maximum activity was retained over temperatures range of 25–60 °C. This indicates that the optimal temperature for GH16B β-agarase activity is slightly higher compared with the temperature (20 °C) at which the *E. coli* transformants are grown to produce recombinant GH16B β-agarase in extracellular soluble forms. The enzymatic activity was observed over a wide pH range of 5.0–8.0 (optimum pH 7.0) (Fig. 5b). The enzyme was stable up to 50 °C and retained ~ 90% and ~ 80% of its maximum activity for 3 and 14 h, respectively; however, most of the enzymatic activity was lost after treatment at 55 °C for 14 h or 60 °C for 3 h, indicating that GH16B β-agarase is a highly thermostable enzyme (Fig. 5c). Although GH16B β-agarase was stable over the pH range of 5.0–8.0, it rapidly became inactive at an acidic pH of ≤ 4.0 and retained ~ 35% activity after 4-h treatment at pH 10.0 (Fig. 5d). These findings suggest that GH16B β-agarase is more stable at alkaline pH than at acidic pH. The kinetic parameters, *Km*, *Vmax*, *kcat*, and *kcat/Km*, of GH16B β-agarase for agarose liquefaction were 14.40 mg/mL, 542.0 U/mg, 576.3 s^−1^, and 4.80 ⨯ 10^6^ s^−1^ M^−1^, respectively (Fig. 5e).

**Fig. 6:**
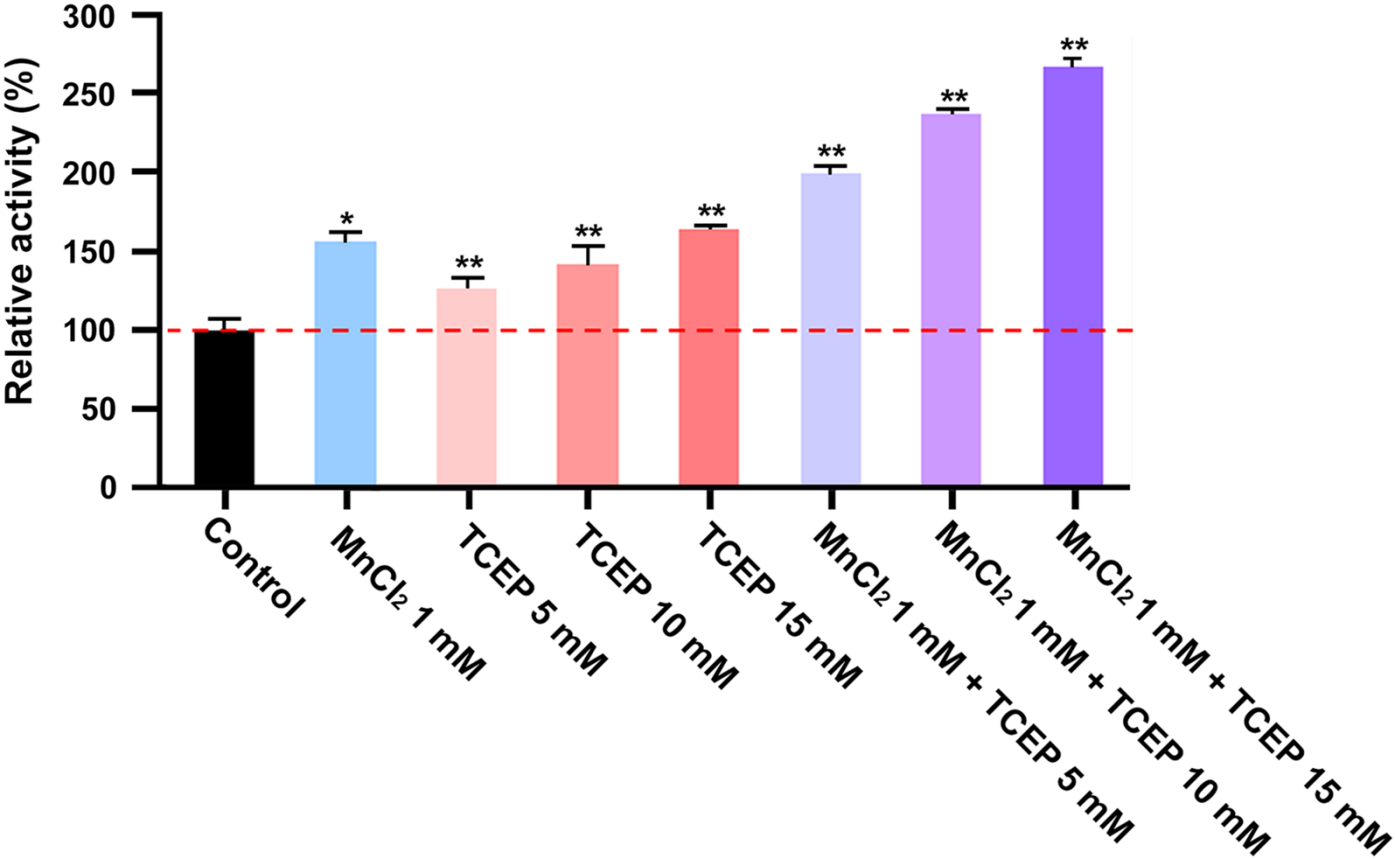
Effects of MnCl_2_ and TCEP on the GH16B β-agarase activity. Individual and cotreatment effects of MnCl_2_ (1 mM) and a sulfhydryl reductant TCEP (5, 10, or 15 mM) on the enzymatic activity were investigated by incubating the purified enzyme (0.8 µg/mL) with 0.4%[w/v] agarose in 50 mM Mcllvaine buffer (pH 7.0) at 50 °C for 30 min. The enzymatic activity measured in the absence of metal ions and TCEP was defined as 100%. *P < 0.05 and **P < 0.01 compared with nontreated control

The GH16B β-agarase activity was enhanced by 1.5–1.6-fold in the presence of 0.5–2.5 mM MnCl_2_ and MnSO_4_, with maximum enhancement in the presence of 1 mM MnCl_2_, indicating that the enzymatic activity depends on Mn^2+^ ions (Table 1). However, CaCl_2_, MgCl_2_, KCl, NaCl, and EDTA at concentrations ranging from 0.5 to 2.5 mM had no significant effect on the enzymatic activity, whereas 2.5 mM CuSO_4_ reduced the enzymatic activity to 74% of that in the control. Furthermore, at a concentration of 5–15 mM, the antioxidant TCEP, which is currently one of the most widely used thiol reducing agents (Burns et al. 1991), dose-dependently enhanced the enzymatic activity, with a maximum enhancement of 1.6-fold (Fig. 6). The enzymatic activity was enhanced by 2.6-fold in the presence of 1 mM MnCl_2_ and 15 mM TCEP, indicating a synergistic effect of Mn^2+^ and TCEP on GH16B β-agarase activity. These findings suggest that Mn^2+^ ions are required for the appropriate enzymatic activity, and the interaction of the enzyme with Mn^2+^ ions may be resistant to the inhibitory chelating action of EDTA.

**Table 1:** Effect of metal ions and other chemicals on GH16B β-agarase activity

Metal ion/chemical (mM)		Relative activity (%)^a^	Metal ion/chemical (mM)		Relative activity (%)^a^
Control		100 (± 1.15)	CaCl_2_	0.5	96 (± 1.80)
				1.0	110 (± 6.85)
				2.5	103 (± 0.90)
MnCl_2_	0.5	125 (± 1.40)	KCl	0.5	103 (± 1.20)
	1.0	163 (± 2.60)		1.0	109 (± 6.41)
	2.5	155 (± 3.00)		2.5	104 (± 3.45)
MnSO_4_	0.5	146 (± 1.95)	CuSO_4_	0.5	103 (± 0.25)
	1.0	156 (± 0.70)		1.0	97 (± 1.45)
	2.5	156 (± 1.90)		2.5	74 (± 1.45)
MgCl_2_	0.5	94 (± 2.80)	TCEP	0.5	100 (± 1.55)
	1.0	107 (± 0.80)		1.0	109 (± 2.85)
	2.5	100 (± 3.55)		2.5	117 (± 6.00)
NaCl	0.5	100 (± 0.30)	EDTA	0.5	95 (± 4.75)
	1.0	109 (± 5.33)		1.0	95 (± 2.02)
	2.5	101 (± 2.25)		2.5	91 (± 4.35)

^a^Values represent the agarase activity (%) expressed as mean ± SD (*n* = 3) relative to nontreated control

### Substrate degradation pattern of GH16B β-agarase

**Fig. 7:**
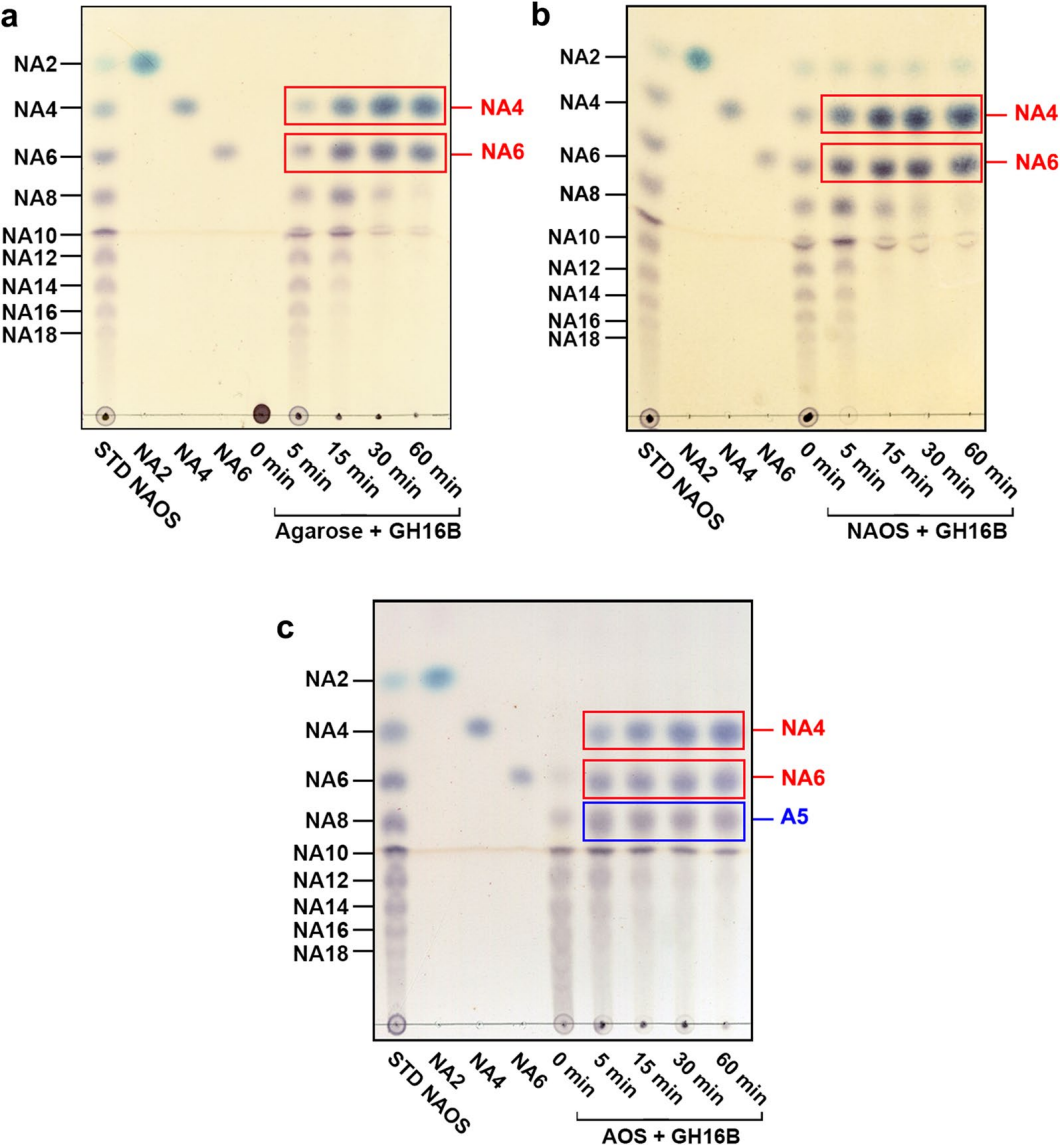
Time-kinetic analysis of GH16B β-agarase-catalyzed hydrolysates of agarose, NAOS, and AOS using thin layer chromatography. **a**–**c** Each substrate (0.4%[w/v] agarose, 1.0%[w/v] NAOS, or 1.0%[w/v] AOS) was treated with the purified enzyme (0.8 µg/mL) in 50 mM Mcllvaine buffer (pH 7.0) at 50 °C for the indicated time. Standard NAOS (STD NAOS) and AOS were prepared as described in “Materials and methods” section. Equivalent amounts of each sample were spotted on TLC plates and developed with *n*-butanol-ethanol-H_2_O (3:2:2[v/v]). Oligosaccharides were visualized as described in “Materials and methods” section. Representative results are shown; two additional experiments yielded similar results

Most GH16 family β-agarases are endo-acting enzymes that hydrolyze agarose to produce NA4/NA6 as end products, although some of them predominantly produce either NA2/NA4 or NA2/NA4/NA6 (Chi et al. 2012; Michel et al. 2006). The hydrolytic products obtained after treating the substrate (agarose, NAOS mixture of NA2–NA18, or AOS) with GH16B β-agarase at different time points were subjected to TLC analysis to determine the substrate degradation pattern. Endolytic degradation of agarose yielded NAOS with various DPs in the early stage and NA4/NA6 as end products in the late stage of the reaction (60 min), according to time-kinetic hydrolysis patterns of GH16B β-agarase catalysis (Fig. 7a). However, NA2 was not detected under these conditions. TLC analysis of the enzymatic hydrolysates of NAOS mixture (NA2–NA18) revealed that NAOS which were as large as or larger than NA12–NA18 were the preferred substrates for producing NA4/NA6 (Fig. 7b). TLC analysis also revealed that an AOS mixture containing various DPs can be degraded by GH16B β-agarase to yield NA4/NA6 as the major product (Fig. 7c). Concurrently, this reaction may produce a similar amount of agaropentaose (A5) that was presumably generated from the nonreducing end of AOS during enzyme-catalyzed AOS digestion.

**Fig. 8:**
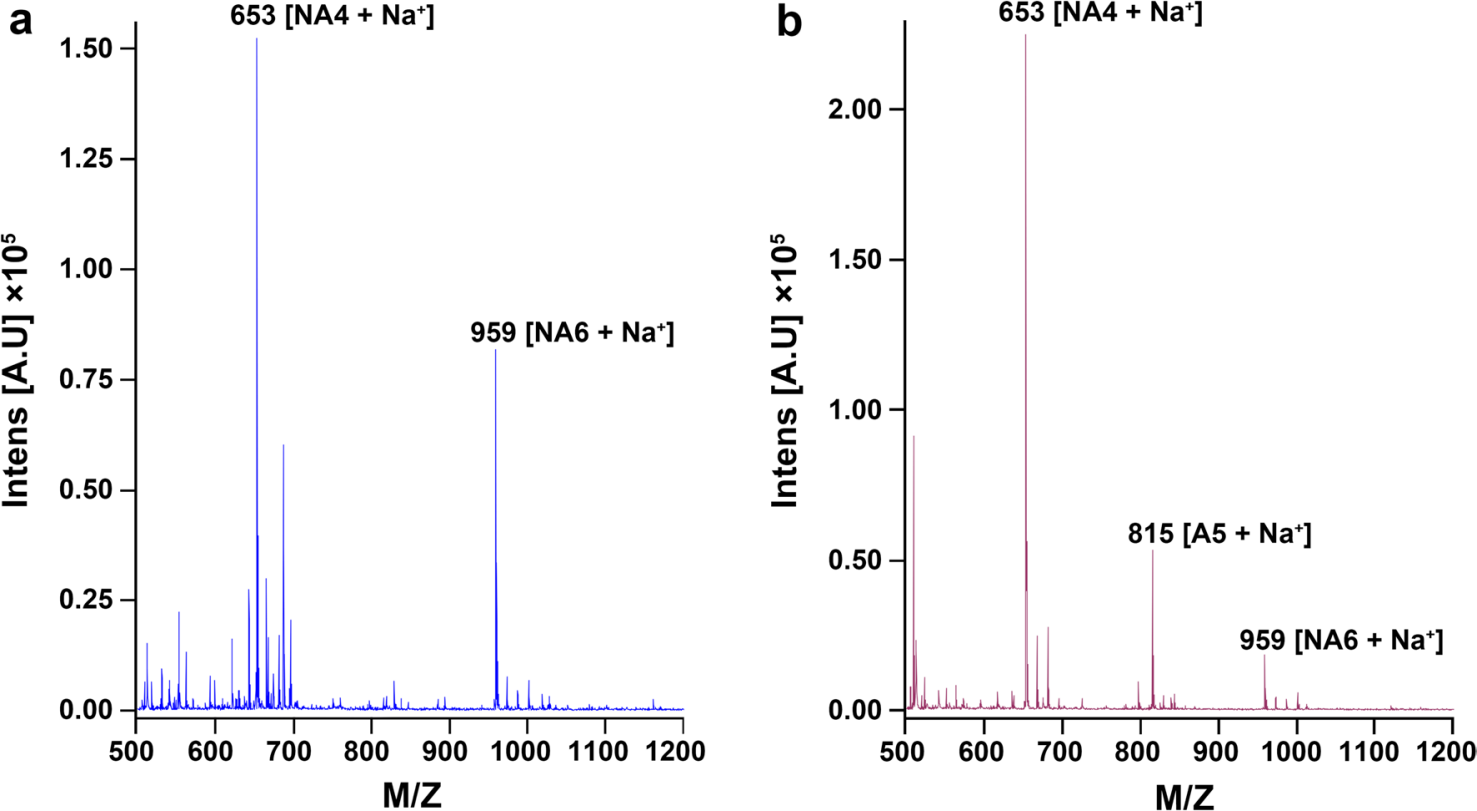
Analysis of GH16B β-agarase-catalyzed hydrolysates of agarose and AOS using MALDI–TOF/TOF MS. **a** For agarose hydrolysis, 0.4%[w/v] agarose was treated with the purified enzyme (0.8 µg/mL) in 50 mM Mcllvaine buffer (pH 7.0) at 50 °C for 60 min. **b** For AOS hydrolysis, 1.0%[w/v] AOS was treated with the purified enzyme (0.8 µg/mL) in 50 mM Mcllvaine buffer (pH 7.0) at 50 °C for 60 min. MALDI–TOF/TOF MS analysis was performed as described in “Materials and methods” section

When the GH16B β-agarase-catalyzed hydrolysate was further analyzed using MALDI–TOF/TOF MS, the detected masses of NA4 ([M+Na]^+^: 653) and NA6 ([M+Na]^+^: 959) corresponded to those calculated from their molecular formulas, as previously described (Lee et al. 2014) (Fig. 8a). Furthermore, MALDI–TOF/TOF MS analysis of the enzyme-catalyzed hydrolysate of AOS revealed that considerable amounts of A5 ([M+Na]^+^: 815), NA4, and NA6 were produced as end products of the reaction (Fig. 8b). These findings confirm that GH16B β-agarase endolytically hydrolyzes agarose into NA4 and NA6, whereas AOS produces not only NA4/NA6 but also A5.

Overall, these findings indicate that recombinant GH16B β-agarase derived from *Cellvibrio* sp. KY-GH-1 can be used to produce NA4 and NA6 in a large quantities from agarose, NAOSs, or AOSs.

### NA4 and NA6 yield upon treatment of agarose with GH16B β-agarase under optimum conditions

**Fig. 9:**
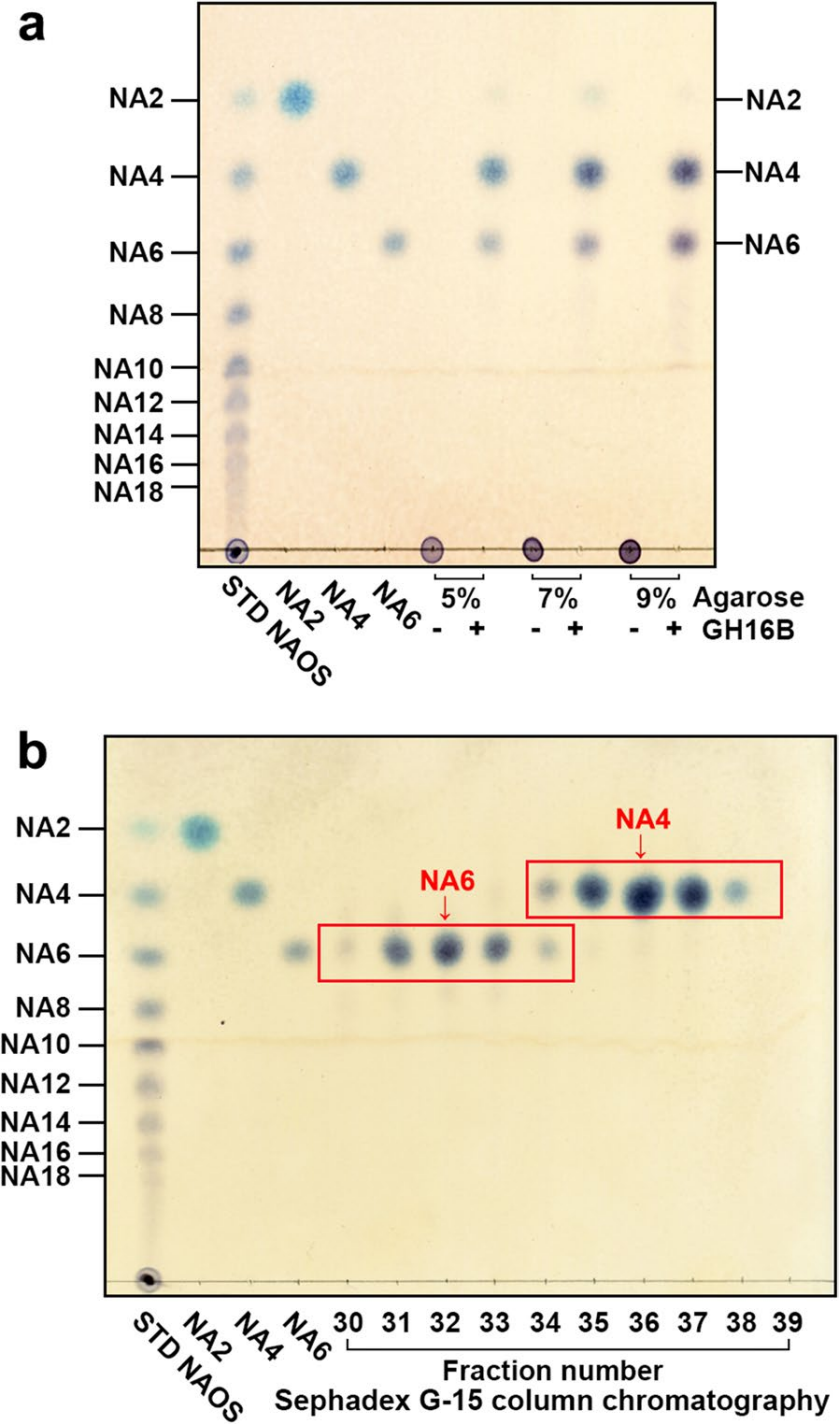
GH16B β-agarase-catalyzed complete hydrolysis of agarose to NA4 and NA6, and purification of NA4 and NA6 from the hydrolysate via Sephadex G-15 column chromatography. **a** After agarose at various concentrations (5.0%[w/v], 7.0%[w/v], or 9.0%[w/v]) in 50 mM Mcllvaine buffer (pH 7.0) was autoclaved, the melted agarose was treated with the purified enzyme (1.6 µg/mL) under optimum reaction conditions (5 mM MnCl_2_, 10 mM TCEP, 50 mM Mcllvaine buffer, pH 7.0) and continuous magnetic stirring at 50 °C for 14 h, the reaction mixtures were subjected to TLC to identify NA4 and NA6 produced from agarose. **b** For purification of NA4 and NA6 from the enzymatic hydrolysate of agarose, 9.0%[w/v] agarose (20 mL) was treated with the enzyme (1.6 µg/mL) at 50 °C for 14 h and then freeze–dried. The dried sample was dissolved in DI water and subjected to Sephadex G-15 column chromatography. Fractions containing NA4 and NA6 were identified using TLC. Representative results are shown; two additional experiments yielded similar results

To further examine whether GH16B β-agarase is efficient in producing NA4/NA6 from agarose, we investigated the maximum concentration of agarose that GH16B β-agarase could efficiently hydrolyze under continuous magnetic stirring at 50 °C, which is a temperature higher than the agarose gelling temperature (~ 40 °C). The hydrolysis products of agarose (5.0%[w/v], 7.0%[w/v], and 9.0%[w/v]) were analyzed using TLC after 14 h of treatment with GH16B β-agarase (1.6 µg/mL) under optimal reaction conditions (1 mM MnCl_2_, 10 mM TCEP, 50 mM Mcllvaine buffer, pH 7.0, 50 °C). The complete hydrolysis of agarose into NA4 and NA6 was achieved up to 9.0%[w/v] agarose (Fig. 9a). Following complete hydrolysis of 9%[w/v] agarose (20 mL) with 1.6 µg/mL GH16B β-agarase for 14 h under optimal reaction conditions, the hydrolysate was freeze–dried. The dried sample was then fractionated via Sephadex G-15 column chromatography with DI water as the mobile phase. In TLC analysis, NA4 was detected in fractions 30–34, and NA6 was detected in fractions 35–39 (Fig. 9b). NA4 and NA6 powders were recovered from the freeze–dried fractions 30–33 and 35–38, respectively. When an enzymatic hydrolysate of agarose (1.8 g) was subjected to Sephadex G-15 column chromatography, ~ 650 mg NA4 and ~ 900 mg NA6 was recovered, representing approximately 85.3% of the theoretical maximum yield.

## Discussion

Herein, we demonstrated that recombinant GH16B β-agarase derived from the agarolytic bacterium *Cellvibrio* sp. KY-GH-1 is produced extracellularly in the *E. coli* expression system and can split the β-1,4-linkage in agarose to yield NA4 and NA6 as end products.

Previously, we observed that the genome of *Cellvibrio* sp. KY-GH-1 contains four putative GH16 β-agarase genes (*β-CvAga16A*, *β-CvAga16B*, *β-CvAga16C*, and *β-CvAga16D*), which encode GH16 β-agarase isozymes that hydrolyze agarose into NA4/NA6 (Kwon et al. 2019). Although these four enzymes were highly expressed in *E. coli* transformants as recombinant His-tagged proteins, only GH16B β-agarase with an N-terminal 22-aa signal sequence was secreted into the culture medium in a soluble form and exerted efficient endolytic hydrolyzing activity, cleaving agarose into NA4/NA6. GH16B β-agarase without the signal sequence was not secreted extracellularly and accumulated primarily in the insoluble inclusion bodies in *E. coli* transformants. Most proteins destined for transport across bacterial cytoplasmic membranes are synthesized with a signal sequence at their N-terminus, which is then removed by a signal peptidase during or immediately after the membrane translocation event (Dalbey et al. 2012; Freudl 2018). However, it remains unclear whether the recombinant GH16B β-agarase secreted from *E. coli* transformants retains the signal sequence. SDS–PAGE and western blot analyses revealed that the overexpression of GH16B β-agarase with the signal sequence in *E. coli* transformants may cause the cells to leak some proteins, such as GH16B β-agarase and several intracellular proteins (e.g., β-galactosidase). However, the mechanism underlying this phenomenon remains unknown.

Alignment of the GH16B β-agarase sequence with its top nine homologs in the NCBI GenBank database revealed that GH16B β-agarase homologs exist in all agar-degrading marine and nonmarine bacteria, suggesting that GH16B β-agarase is critical for the bacterial agar-degrading enzymatic machinery. Furthermore, as the enzymatic properties of none of these nine homologs were examined, the current data on GH16B β-agarase may provide insights into GH16 family β-agarases with significant homology to GH16B β-agarase.

KY-GH-1 GH16B β-agarase showed comparable *Km* and *Vmax* values (14.40 mg/mL and 542.0 U/mg) for agarose liquefaction to those of other reported GH16 β-agarases, including GH16 β-agarase (2.59 mg/mL and 275.48 U/mg) from *Cellulophaga omnivescoria* W5C (Ramos et al. 2018), GH16 β-agarase (3.78 mg/mL and 11,400 U/mg) from *Catenovulum agarivorans* YM01 (Cui et al. 2014), GH16 β-agarase (3.9 mg/mL and 909.1 U/mg) from *Agarivorans* sp. LQ48 (Long et al. 2010), GH16 β-agarase (28.33 mg/mL and 88.25 U/mg) from *Pseudoalteromonas hodoensis* H7 (Park et al. 2015), GH16 β-agarase (7.7 mg/mL and 18.3 U/mg) from *Saccharophagus degradans* 2-40^T^ ((Kim et al. 2017a), GH16 β-agarase (4.8 mg/mL and 517 U/mg) from *Microbulbifer* sp. JAMB-A94 (Ohta et al. 2004), GH16 β-agarase (2.33 mg/mL and 101 U/mg) from *Vibrio* sp. strain PO-303 (Dong et al. 2007). Furthermore, GH16B β-agarase appeared to be more catalytic than other related enzymes in terms of *kcat* and *kcat/Km* values (576.3 s^−1^, and 4.80 × 10^6^ s^−1^ M^−1^, respectively) for agarose liquefaction.

Unlike the marine-origin β-agarase activity, which generally requires Mg^2+^ and Na^+^ ions (An et al. 2018; Han et al. 2016; Xie et al. 2013), MgCl_2_ or NaCl had no significant effect on GH16B β-agarase activity. Instead, the presence of Mn^2+^ or TCEP appeared to boost the enzymatic activity in a dose-dependent manner. Mn^2+^ and TCEP had a synergistic effect on the enzymatic activity, with up to 2.6-fold increase in the presence of 1 mM MnCl_2_ and 15 mM TCEP. The Mn^2+^/TCEP-dependent enhancement of GH16B β-agarase activity was comparable to that of *Cellvibrio* sp. KY-GH-1 GH50A β-agarase (Kwon et al. 2020) or GH117A α-NABH (Jang et al. 2021). These previous and current findings indicate that the copresence of Mn^2+^ and TCEP increases the activities of all three enzymes, which are essential components of the agarose-degrading enzyme machinery involved in the degradation of agarose into L-AHG and D-Gal in KY-GH-1 strain.

The recombinant GH16B β-agarase from *Cellvibrio* sp. KY-GH-1 is highly thermostable up to 50 °C. Previously, a recombinant GH16 β-agarase YM01-3, obtained from the marine bacterium, *C. agarivorans* YM01, was reported to exhibit optimal activity at 60 °C and retained ~ 80% activity at 50 °C for 1 h; moreover, it exhibited higher thermal stability than other reported GH16 β-agarases (Cui et al. 2014). These current and previous results suggest that although the optimum temperature for GH16B β-agarase activity (50 °C) is lower than that for GH16 β-agarase YM01-3 activity (60 °C), the thermal stability of GH16B β-agarase is significantly higher at 50 °C. Because the sol-state agarose is more feasible for the catalysis of β-agarases than the gel-state agarose (Fujii et al. 2000; Kim et al. 2017a) and 5–10%[w/v] melted agarose requires a gelling temperature of approximately 40–48 °C, the optimum temperature (50 °C) and thermostability (stable at 50 °C for 14 h) of GH16B β-agarase can facilitate its application in efficient enzymatic liquefaction of melted agarose at concentrations of up to 10%[w/v] and its conversion into NAOSs with low DPs.

Consistent with previous studies showing that GH16 family β-agarases endolytically hydrolyze agarose to produce NA4 and NA6 as major end products (Cantarel et al. 2009; Chi et al. 2012; Michel et al. 2006), TLC analysis of 0.8 µg/mL GH16B β-agarase-catalyzed hydrolysates of either 0.4%[w/v] agarose or 1.0%[w/v] NAOS, obtained after incubation at 50 °C for 1 h, showed only NA4 and NA6 as end products. On the contrary, TLC analysis of 1.6 µg/mL GH16B β-agarase-catalyzed hydrolysates of 5%[w/v], 7%[w/v], and 9%[w/v] melted agarose, obtained after incubation at 50 °C for 14 h under continuous magnetic stirring, reveled complete agarose digestion into the end products NA4/NA6, with the detection of only trace amounts of NA2. This finding suggests that high concentrations of GH16B β-agarase can further hydrolyze NA6 into NA4 and NA2. Notably, GH16B β-agarase could degrade an AOS mixture with various DPs, which were obtained via the mild acid-hydrolysis of agarose (Kwon et al. 2020), to yield NA4, NA6, and A5 as end products.

Under optimal reaction conditions (1 mM MnCl_2_, 10 mM TCEP, 50 mM McIlvaine, pH 7.0, 50 °C), treatment with 1.6 µg/mL GH16B β-agarase for 14 h under continuous magnetic stirring efficiently hydrolyzed up to 9.0%[w/v] melted agarose into NA4 and NA6. When NA4 and NA6 were purified from the hydrolysates of 9%[w/v] agarose via size-exclusion column chromatography, the recovery of NA4 and NA6 was ~ 85% of the theoretical maximum yield, suggesting that recombinant GH16B β-agarase may be a robust enzyme for industrial use in enzymatic liquefaction of agarose and subsequent mass production of NA4/NA6.

In summary, the recombinant GH16B β-agarase. which was expressed extracellularly in the *E. coli* expression system, could efficiently hydrolyze up to 9%[w/v] agarose in an endo-acting manner to produce NA4 and NA6. The optimal temperature and pH for the enzymatic activity for agarose hydrolysis were 40–50 °C and 7.0, respectively. The enzyme was stable at a temperature of up to 50 °C and pH of 5.0–8.0. In the presence of 1 mM MnCl_2_ and 15 mM TCEP, the enzymatic activity was enhanced by 2.6-fold. The *Km*, *Vmax*, *kcat*, and *kcat/Km* values for agarose liquefaction were 14.40 mg/mL, 542.0 U/mg, 576.3 s^−1^, and 4.80 × 10^6^ s^−1^ M^−1^, respectively. The thermal stability and endolytic action of GH16B β-agarase suggest that it is useful for mass production of NA4 and NA6 via one-step agarose liquefaction at 50 °C, which is higher than the gelling temperature of melted agarose. Alternatively, agarose liquefaction using GH16B β-agarase can be combined with a potent exolytic GH50 family β-agarase for efficient agarose degradation into NA2 or can be combined with the exolytic GH50 family β-agarase as well as α-NABH for efficient agarose degradation into the monosaccharides L-AHG and D-Gal. Finally, because the enzymatic properties of none of the nine GH16B β-agarases with 50.8−98.8% homology have been examined to date, current data on GH16B β-agarase from *Cellvibrio* sp. KY-GH-1 may provide insights into β-agarases exhibiting significant homology with GH16B β-agarase.

## Data Availability

The authors promise the availability of data and materials.
